# Optical fiber sensor for water velocity measurement in rivers and channels

**DOI:** 10.1038/s41598-024-64202-5

**Published:** 2024-06-11

**Authors:** Armando Rodriguez, Pedro Dieguez, Jose Carlos Urroz, Mikel Bravo, Javier Lopez, Manuel Lopez-Amo

**Affiliations:** 1 Department of Electrical, Electronic and Communication Engineering, Universidad Publica de Navarra, 31006 Pamplona, Spain; 2 Institute of Smart Cities (ISC), Universidad Publica de Navarra, 31006 Pamplona, Spain; 3 Engineering Department, Universidad Publica de Navarra, 31006 Pamplona, Spain; 4 School of Industrial and ICT Engineering, Universidad Publica de Navarra, 31006 Pamplona, Spain

**Keywords:** Coatings, Elastic coefficient, Fiber optic sensors, Strain, Water velocity, Optical sensors, Mechanical engineering

## Abstract

In this work, optical fiber Bragg grating sensors were used to measure water velocity and examine how it was distributed in open channels. Several types of coatings were incorporated into the design of the sensors to examine their effects on the strain that the fibers experienced as a result of the water flow. Due to their low elastic coefficient, which reduced the hysteresis, the results indicated that the aluminum- and acrylate-coated fibers had the best performance. ANSYS-CFX V2020 R2 software was used to model the strain encountered by the fibers under various flow rates to assess the performance of the FBG sensors. The calculations and actual data exhibited good convergence, demonstrating the accuracy of the FBG sensors in determining water velocity. The study illustrated the usability of the proposal in both scenarios by contrasting its application in rivers and channels.

## Introduction

The accurate measurement of water velocity is essential for many applications, such as environmental monitoring, hydraulic engineering, and water resource management. In recent years, optical fiber sensors have emerged as a promising technology for water velocity measurement because of their high sensitivity, fast response time, and ability to measure velocity at multiple points simultaneously. Unlike traditional measurement techniques that rely on mechanical components that can degrade over time, fiber optic sensors are immune to electromagnetic interference and can operate in harsh environments. As a result, there has been growing interest in the development and application of fiber optic sensors for water velocity measurement.

Effective water resource management requires continuous monitoring of streamflow to plan and operate water supply and distribution systems. To obtain a complete record of streamflow, systematic measurements must be taken on an ongoing basis. A hydrograph provides a visual representation of discharge values over time at a specific point in a river or channel. To construct a hydrograph, measurements of the water surface height (stage) are taken and then converted into flow using a stage-discharge curve. Discharge, which is the volume of water flowing through a channel, can be determined by measuring the velocity-area ratio at stream gauging stations.

Typically, electronic or mechanical devices are used to measure the velocity of water in channels. The classical flow meters used are a Venturi flowmeter, a Pitot flowmeter, an orifice plate flowmeter or rotameter. These flowmeters cause effects of upstream conditions, and their accuracy is approximately 5%^1,2^. However, flowmeters based on new technologies (Coriolis, ultrasonic, vortex, electromagnetic, etc.) generally offer high accuracy, a wide range of flow rates, and nonintrusive flow measurements. A recent review of these sensors can be found in^3^. It should be noted that some of them, the most accurate ones, can exceed $10 000 in price, such as the electromagnetic flowmeter used in Section "Results and discussion" of this paper.

As mentioned before, due to their small size and resistance to electromagnetic interference (EMI), which can be attributed to the use of dielectric materials, optical fiber sensors are desirable for monitoring water velocity at point locations without altering the water distribution within canals. Additionally, because they are fabricated using silica, they can be used in liquids and can be interrogated from some kilometers away from the measuring equipment stations^4^. Three main sensing methods have been used for the development of optical fiber sensors for flow measurements: intensity-modulated fiber sensors^5^, wavelength-modulated fiber sensors^6^, and distributed and phase-modulated optical fiber sensors^7^. One particular type of optical fiber sensor that is well suited for point measurements is the fiber Bragg grating (FBG) sensor^8,9^. Previous research has demonstrated the efficacy of FBGs in measuring flow rates within enclosed conduits^6,10^ as well as open structures and applying interferometric techniques^11,12^. In^10^ demonstrated the feasibility of measuring water velocities higher than 4 m s^−1^ using FBGs in pipes. These high velocities are very difficult to find in real open channels and rivers, thus FBGs are perfectly suited for this application, and their measurement capabilities in both low-speed and high-velocity channels will be demonstrated. With a single FBG interrogator, as used in Section "Results and discussion" of this paper, tens of FBGs can be simultaneously interrogated^13^. These sensors can be placed in different locations of the channel or river or at different heights, as shown in Section "Results and discussion" of this paper.

In this work, FBG fiber optic sensors for measuring water velocity using two different coatings were theoretically analyzed and experimentally developed, one of which was especially hardened to prevent problems in natural water environments. The two kinds of sensors were computationally simulated to evaluate their performance and limitations. Firstly, a general outline is provided of the underlying theories of fiber optic FBG sensing and the difficulties in precisely monitoring water velocity. The two selected coatings for the sensors are then discussed, along with the computer simulations we conducted to assess their performance. Afterwards, the theoretical models and simulations were experimentally verified in an open channel in the laboratory. Finally, using an electromagnetic water flow meter to validate the sensors, the performance of FBG fiber sensors, especially those designed for water flow measurements in open channels and rivers, was tested for the first time to our knowledge under high-speed rates, and the results showed good measurement performance and robustness.

### Sensor material analysis: theory and computational simulation

To calibrate the sensor, theoretical calculations, computational simulations and tests were performed in the laboratory, channels and rivers. Although other covering materials have been assessed on the fiber and a comparative study is discussed in^10^, the acrylate covering will be utilized as a reference in the following evaluations. Acrylate is a well-known and less expensive material used in fiber coatings, providing great flexibility and ease of use in laboratory and field applications. All the performed tests were cross-correlated with the temperature effect by placing an FBG with a metallic cover in line with the flow direction. The reinforced coating prevents straining of the sensor and thus measures only the temperature variation in the water.

The velocity of the water applied a hydrodynamic force on the fiber optic sensor wire, deforming it or simply stretching it. This deformation can be calculated theoretically via flow dynamics and resistance of materials and computationally via computational fluid dynamics (CFD) and structural simulation programs. In addition, this deformation can be measured by the sensor by changes in wavelength. The aim of this section, subdivided into two parts, is to calculate the deformation of the sensors both theoretically and computationally. Afterwards, these deformations are compared with the values measured in the laboratory and in outdoor application scenarios (channels and rivers).

### Laboratory study of channels

**Figure 1:**
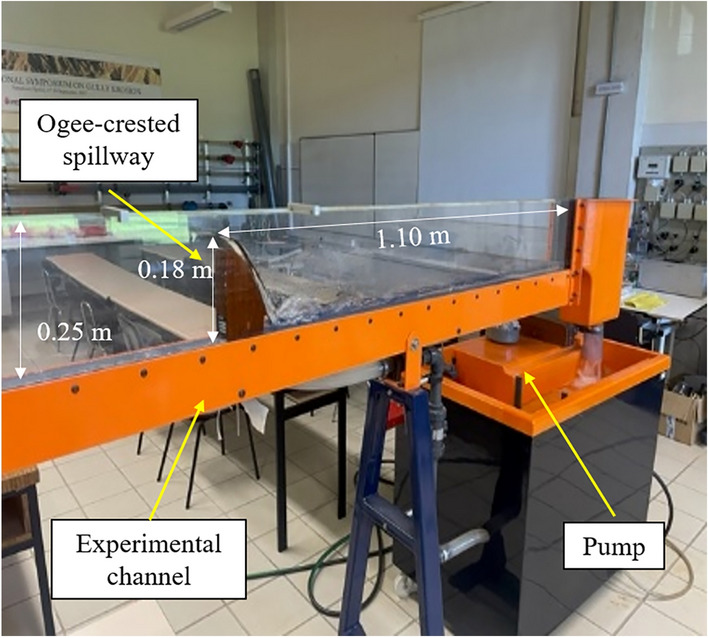
Channel in Laboratory with an ogee-crested spillway for water lever control.

For the setting up of the material and tests, as depicted in Fig. 1, a tilting flume was used (dimensions 4.960 m long, 0.250 m high and 0.077 m wide) with a 0.180 m high obstacle (an ogee-crested spillway) from Armfield Limited that drives water flow rates between 1.5 and 8.5 m^3^ h^−1^.

Considering that the water flow rate (m^3^ s^−1^) is the product of the water velocity (m s^−1^) by the channel section (m^2^), for an estimation of the order of magnitude of the water velocities obtainable in the channel, the ratio between maximum flow (8.5 m^3^ h^−1^ or 8.5/3600 m^3^ s^−1^) and section (0.250 m high by 0.077 m wide), that is, (8.5/3600)/(0.250, 0.077) = 0.123 m s^−1^ is enough. This value indicates that the velocities to be measured are low, even for the maximum driven flow. In addition, the relatively small thickness of the channel (0.077 m) has two implications: conventional water velocity meters do not fit into it, and there is a velocity gradient toward the walls of the channel. Both circumstances force the computational calculation of velocities and sensor deformation.

**Figure 2:**
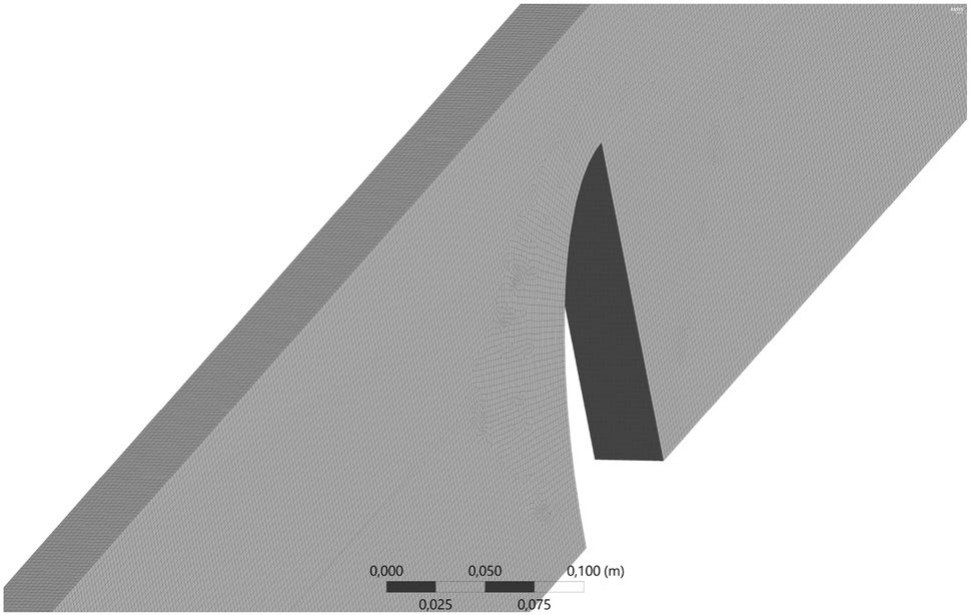
Simulated 3D mesh of the fluidic domain.

To assess the water velocity measured in the laboratory channel, a set of computational simulations were performed using the ANSYS-CFX V2020 R2 program^14^. This program includes ANSYS-Mesh, a powerful software that divides the three-dimensional fluidic domain of water into millions of small finite volumes. A three-dimensional mesh was created formed by 2 805 888 elements (1 664 prisms and 2 804 224 hexahedrons), as shown in Fig. 2. To reduce the computational time, only half of the domain is considered because of the symmetry condition with respect to the longitudinal vertical plane. To simplify the simulation process, we assumed an isothermal water flow and used the standard *k-ε* turbulence model. The fluid buoyancy model accounts for density differences but does not include interphase transfer or surface tension effects.

This powerful finite volume program allows to simulate each of the tested flow rates (1.5, 2.5, 3.5, 4.5, 5.5, 6.5, 7.5 and 8.5 m^3^ h^−1^). Depending on the flow rate, the mass and momentum equations can be solved with a variable number of iterations ranging from 325 to 1023. After running the simulations, it can be seen that the convergence level is very high: the maximum imbalance mass water is 4%, and the RMS residual is 10^−5^.

**Figure 3:**
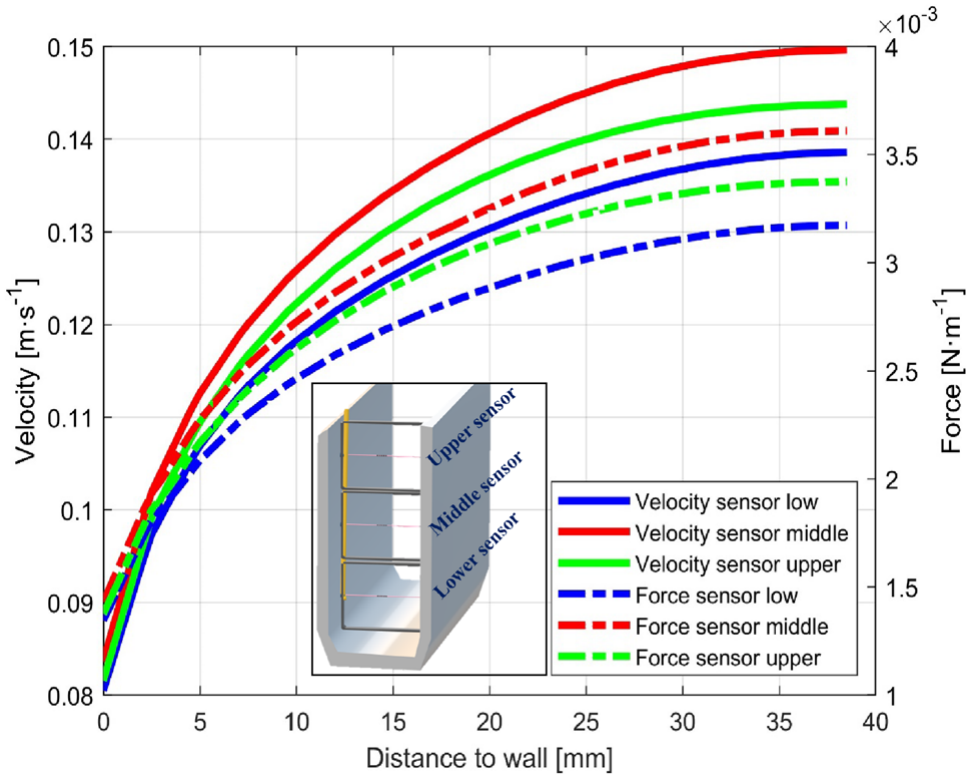
Water velocity and forces on the optical fiber sensor.

In the postprocessing of the simulations, the gradient of velocities and forces exerted by the water on the sensor, as a function of the distance to the wall, is determined at the position where the sensors will be located, 1.00 m upstream of the obstacle, at heights of 30 mm, 103 mm and 175 mm. In Fig. 3**,** these values are shown for a water flow rate of 8.5 m^3^ h^−1^.

To calculate the forces exerted on the sensor, the outer sensor diameter datum *D* = 180 μm is necessary, and for the calculation of the deformations, the stiffness modulus datum *EA* = 860 N is necessary. The morphology and properties of the sensors used are detailed in the experimental section.

The forces generate a deformation on the sensor. The fiber deformations were also calculated via computational simulation with ANSYS Mechanical APDL, which is a finite element program that can be used to carry out static and dynamic structural analyses. The LINK180 element type from the ANSYS library was used. This 3D spar is useful for a variety of engineering applications. The element can be used to model cables and wires. The element is a uniaxial tension–compression element with three degrees of freedom at each node: translations in the nodal *x*, *y*, and *z* directions. Tension-only (cable) is supported.

A simulation for each water flow rate was performed. The wire was considered, from its junction to the pipe to the pipe axis. As boundary conditions, zero displacements were imposed at the junction of the wire to the pipe, and only longitudinal displacement along the axis of the pipe was imposed at the other end. The applied forces are the forces from Fig. 3, which are distributed along the axis of the wire. Table 1 shows the results of the calculations.

**Table 1:** Computational deformation of the optical fiber sensors.

Vol flow (m^3^ h^−1^)	1.5	2.5	3.5	4.5	5.5	6.5	7.5	8.5
Lower s. (με)	0.06	0.69	1.65	2.85	4.42	6.01	7.55	9.40
Middle s. (με)	0.09	0.86	2.02	3.50	5.24	7.09	8.78	10.73
Upper s. (με)	0.29	0.94	1.85	3.12	4.74	6.26	8.12	10.08

### Study of wide channels and rivers

The gradient of water velocity in a wide channel or river is notably different from that in a laboratory study. Because of the large width (meters or even kilometers in rivers) compared to the width of the laboratory channel (0.077 m), no velocity gradient can be considered; acting on all sensor points has a constant water velocity. Moreover, in this case, it is possible to use conventional high-precision water velocity meters.

The force exerted by a fluid stream on a long perpendicular cylinder is evaluated on the basis of the well-known fluid dynamics formula:

1 $$F_{D} = 0.5 \cdot C_{D} \cdot \rho \cdot A \cdot V^{2}$$

where *F*_*D*_ is the drag force, *A* is the projected area of the cylinder perpendicular to the direction of the velocity *V* of the stream and *C*_*D*_ is the drag coefficient. Considering the projected area of the cylinder *A* = *D* *L* (diameter multiplied by cylinder length), the force *p* applied on the unit length of the cylinder can be determined by the expression:

2 $$p=0.5\cdot {C}_{D}\cdot \rho \cdot D\cdot {V}^{2}$$

The drag coefficient *C*_*D*_ in the case of cylindrical bodies is evaluated in^15^ as a function of the Reynolds number, *R*_*e*_, as follows:

3 $${R}_{e}=(V\cdot D\cdot \rho )/\mu$$

where *V* is the velocity of the flow, *D* is the outer diameter of the cylinder, and *ρ* and *µ* are the density and dynamic viscosity of the fluid, respectively, at its temperature.

**Figure 4:**
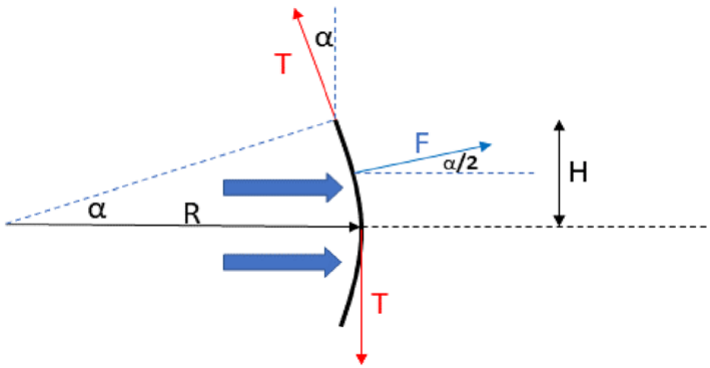
Diagram of forces on the optical fiber sensor.

The fiber optic wire, depicted in Fig. 4**,** is deformed due to the hydrodynamic force *p* applied from left to right (the force distributed on the wire is not shown in the figure). It is assumed that this distributed force per unit length of wire *p* is constant along the wire; thus, the wire is deformed according to an arc of circumference.

The previous picture shows the equilibrium of forces on the upper semi arc, where *T* is the force at any section of the wire, which is shown in red at its junctions to the pipe and axis. *R* is the radius of curvature (to be calculated) of the wire deformation, *H* is half the distance between the two fastenings, *p is* the force per unit length on the wire, *F is* the resultant force on the upper semi arc, and *α* is the angle formed by the reaction force *T* and the undeformed sensor.

As a result of the equilibrium of forces in the direction of water flow, then:

4 $$T\cdot \mathit{sin}\alpha -F\cdot \mathit{cos}(\alpha /2)=0$$

As a result of the equilibrium of forces in the normal direction of water flow, then:

5 $$F\cdot \mathit{sin}(\alpha /2)+T\cdot cos\alpha -T=0$$

This expression is not useful because it is a linear combination of (Eq. 4). This can be easily checked by dividing the last expression by equation (Eq. 4), which gives the trivial equivalence:

6 $$\mathit{tan}(\alpha /2)=\left(1-cos\alpha \right)/sin\alpha$$

In (Eq. 4), the stress is:

7 $$T=EA\cdot \varepsilon$$

where *EA* is the wire rigidity obtained from its geometry and materials. The strain is:

8 $$\varepsilon =\left[\left(\alpha \cdot R-H\right)/H\right]+{\varepsilon }_{0}$$

where *ε*_*0*_ is the initial strain. The force *F* due to the dynamic pressure of water is:

9 $$F=p\cdot \alpha \cdot R$$

From Fig. 4**,** $\sin \alpha = H/R$, then:

10 $$\text{cos}\left(\alpha /2\right)=\sqrt{\left(1+\sqrt{{1-\left(H/R\right)}^{2}}\right)/2} , \forall \alpha \in {\mathbb{R}},0\le \alpha \le \frac{\pi }{2}$$

By making the corresponding substitutions of Eqs. (7), (8), (9) and (10) in Eq. (4) simplifications, the new equation obtained from the equilibrium of forces is:

11 $$EA\left\{\frac{\left[\mathit{arcsin}\left(\frac{H}{R}\right)\right]R-H}{H}+{\varepsilon }_{0}\right\}\frac{H}{R}-p\left[\mathit{arcsin}\left(\frac{H}{R}\right)\right]R\sqrt{\frac{1+\sqrt{1-{\left(\frac{H}{R}\right)}^{2}}}{2}}=0$$

In the experimental validation section, the values of *EA* = 860 N, *H* = 0.08 m and ${\varepsilon }_{0}$ = 2.2840 10^−4^ are justified. Each water velocity implies a force per unit length of wire *p,* which causes the value of *R* to change. The values of *p* were previously calculated from (Eq. 2). This equation (Eq. 11) is transcendental in *R* and is solved by an iterative calculation programmed in MATLAB.

Once *R* is obtained, the calculation of the deformation *ε* is straightforward, corresponding to the value of the expression:

12 $$\left(\frac{\alpha R}{H}-1\right)\text{ or }\left\{\left[arcsin\left(\frac{H}{R}\right)\right]\left(\frac{R}{H}\right)-1\right\}$$

In Table 2, theoretical values obtained from the solution to (Eq. 11) are shown for several water current velocities. Column 1 corresponds to the water velocity *V* (m s^−1^). Referring to the Reynolds number formula (Eq. 3), *D* is the outside diameter of the optical sensor wire, *D* = 250 μm for the acrylate-coated wire but *D* = 180 μm for the aluminum-coated wire (next experimental validation section), *ρ* and *µ* are the density and dynamic viscosity of water at 290 K, *ρ* = 998.8 kg m^−3^ and *µ* = 1.084 10^−3^ kg m^−1^ s^−1^, respectively^16^. Columns 2, 3 and 4 correspond to the calculated values of the Reynolds number, drag coefficient *C*_*D*_ and force per unit length applied by the circulating water on the wire *p* (N m^−1^), respectively. Column 5 corresponds to the radius *R* (m), which is the solution of the force balance equation for each water velocity. Column 6 is the value of the elastic strain *ε* (without the summand *ε*_*0*_ and multiplied by 10^6^). Additionally, columns 7 and 8 show the force *T* (N) in the wire and the angle *α* (°).

**Table 2:** Theoretical data calculated from (Eq. 11).

	1	2	3	4	5	6	7	8
	V(m s^−1^)	R_e_	C_D_	p(N m^−1^)	R(m)	*ε*(*με*)	T(N)	*α*
Acrylate coated	0.05	12	2.804	0.0009	224.224	0.02	0.196	0.02
	0.25	58	1.481	0.0116	17.248	3.59	0.199	0.27
	0.50	115	1.305	0.0407	5.547	34.67	0.226	0.83
	0.75	173	1.243	0.0873	3.245	101.33	0.283	1.41
	1.00	230	1.211	0.1511	2.374	189.36	0.359	1.93
	1.25	288	1.190	0.2321	1.918	290.18	0.445	2.39
	1.50	346	1.170	0.3287	1.638	397.99	0.538	2.80
	1.75	403	1.150	0.4397	1.445	511.56	0.636	3.17
	2.00	461	1.130	0.5643	1.304	628.36	0.736	3.52
Aluminum coated	0.05	8	3.279	0.0007	266.300	0.02	0.196	0.02
	0.25	41	1.616	0.0091	21.817	2.24	0.198	0.21
	0.50	83	1.373	0.0309	6.971	21.95	0.215	0.66
	0.75	124	1.292	0.0653	3.917	69.53	0.256	1.17
	1.00	166	1.250	0.1124	2.792	136.89	0.314	1.64
	1.25	207	1.224	0.1719	2.222	216.17	0.382	2.06
	1.50	249	1.210	0.2447	1.871	304.96	0.458	2.45
	1.75	290	1.190	0.3276	1.640	397.01	0.537	2.80
	2.00	332	1.176	0.4227	1.469	494.95	0.621	3.12

## Experimental setup

As previously stated, several optical fiber coatings were used during the implementation of the suggested measuring approach. The acrylate material utilized was the same as that used for actual communications fibers. To strengthen the system without compromising the flexibility of the fibers, aluminum alloy-coated optical fibers from Technica S.A. were utilized. According to the manufacturer’s specifications, aluminum has been proven to have high resistance to corrosion and rust in high-humidity environments. The acrylate coating showed high interaction with suspended particles in the water flow, adhering to the fiber surface and generating sail effect, thus, the hysteresis in the results increased, as demonstrated in^10^. In the case of aluminum coating, there is no high impact on the measurement because the hysteresis is reduced due to the metal coating and the low plasticity coefficient of the material. However, to avoid any related effects, maintenance programs are often designed for long-term measurement tasks to help solve calibration and sensibility in situ issues.

The experiments were performed under constant temperature conditions, offsetting the sensitivity of the FBGs to this magnitude. In all scenarios, the Micron Optics® SM-130 Optical Sensor Interrogator (OSI) was used, and the sampling rate was set to 1 kHz—the highest it can afford—to acquire data from sensors. The use of custom-made software running on a PC allows adequate statistical analysis to be computed and carried out to correctly establish a relationship between the water flow rate and the strain applied to the fiber.

### Study of wide channels and rivers

**Figure 5:**
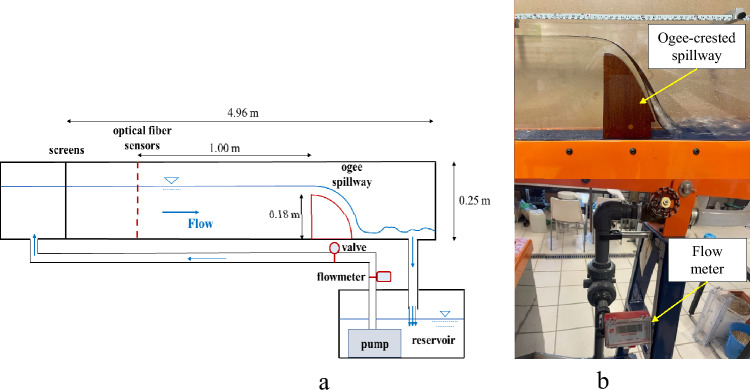
(**a**) Graphical scheme of the experimental installation of the test flume. (**b**) Details of the test flume including ogee-crested spillway and a commercial flow meter from FSL.

To set up the material and tests, the optical FBG sensors in a test flume were checked. The test flume is an open channel. Its dimensions have been described above, and it has an adjustable tilt angle. Figure 5a) shows a schematic of the open channel and the experimental setup, including the optical FBG sensors and the ogee spillway used to raise the water surface (Fig. 5b).

The open channel has a water recirculation system in which the flow is supplied from a tank with a pump. The gratings are placed upstream of the channel to avoid waves and to obtain uniform flow conditions along the channel. The measurement procedure was carried out by driving a stream of water through the channel via a pump and controlling the flow with a control valve. The discharge readings were taken from a BP200F flow meter, manufactured by FSL, which is present in the test flume. The flow rate was increased at 1 m^3^ h^−1^ intervals, from 0.5 m^3^ h^−1^ to 8.5 m^3^ h^−1^, the maximum possible at the pump.

**Figure 6:**
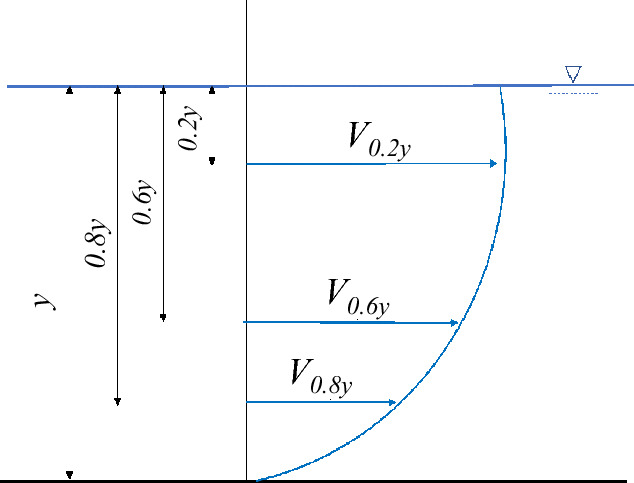
Free-stream water velocity vertical distribution.

In open channel flows, the velocity is distributed unevenly throughout a cross section (see Fig. 6). In comparison, the lowest values are found at bed level, while the highest values are measured at middle heights and at distances near the water's surface. This distribution depends on the tilt angle of the water channel. Hence, it is very useful to place many sensors at various heights. It is possible to calculate the distribution along the section by measuring various flow values and comparing their correlation.

**Figure 7:**
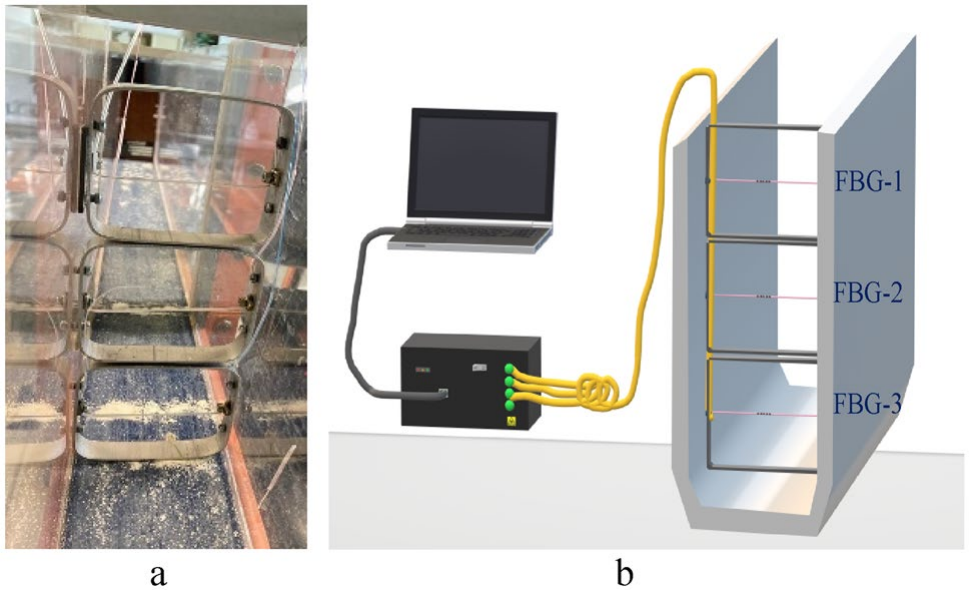
Flow measurement (**a**) arrangement and (**b**) setup for water channels using 3 optical fiber FBG sensors placed at different heights.

Figure 7 shows and describes the designed setup. A pump and a valve allow the flow rate to be set. To increase the level of the water and observe the impact of dragging currents, various pieces of wood can be positioned, as was simulated. A flow meter is used to collect the flow readings, which are correlated with the data acquired via the OSI.

The cross section of the channel was divided into three parts to facilitate the estimation of the water velocity distribution. Each of them contains a steel frame whose dimensions are adjusted to the width of the section. These steel frames contain fibers with FBGs whose sensitive area is located in the center.

Using the same procedure as during the installation in pipes^10^, the sensors were prestressed. In this case, initial deformations of 253 µε for the acrylate-coated fiber and 257 µε for the aluminum-coated fiber were applied.

The bottom sensor (yellow) is reflective at 1535 nm, the intermediate sensor (red) is reflective at 1563 nm, and the upper sensor (blue) reflects at 1549 nm. The wavelengths of the sensors were selected to be spectrally separated from one another. The prestrained fiber specifications and position in the setup are also described in Table 3.

**Table 3:** Sensor arrangement description in independent holders.

λ (nm)	λ (idle) (nm)	λ (placed in holder) (nm)	Pre-strained (in holder) (µε)	Position from bottom (mm)
**1549**	1549,478	1549,654	176	175
*1535*	1535,097	1535,358	261	30
1563	1563,025	1563,685	660	103

### In-field installation in open channels and rivers

**Figure 8:**
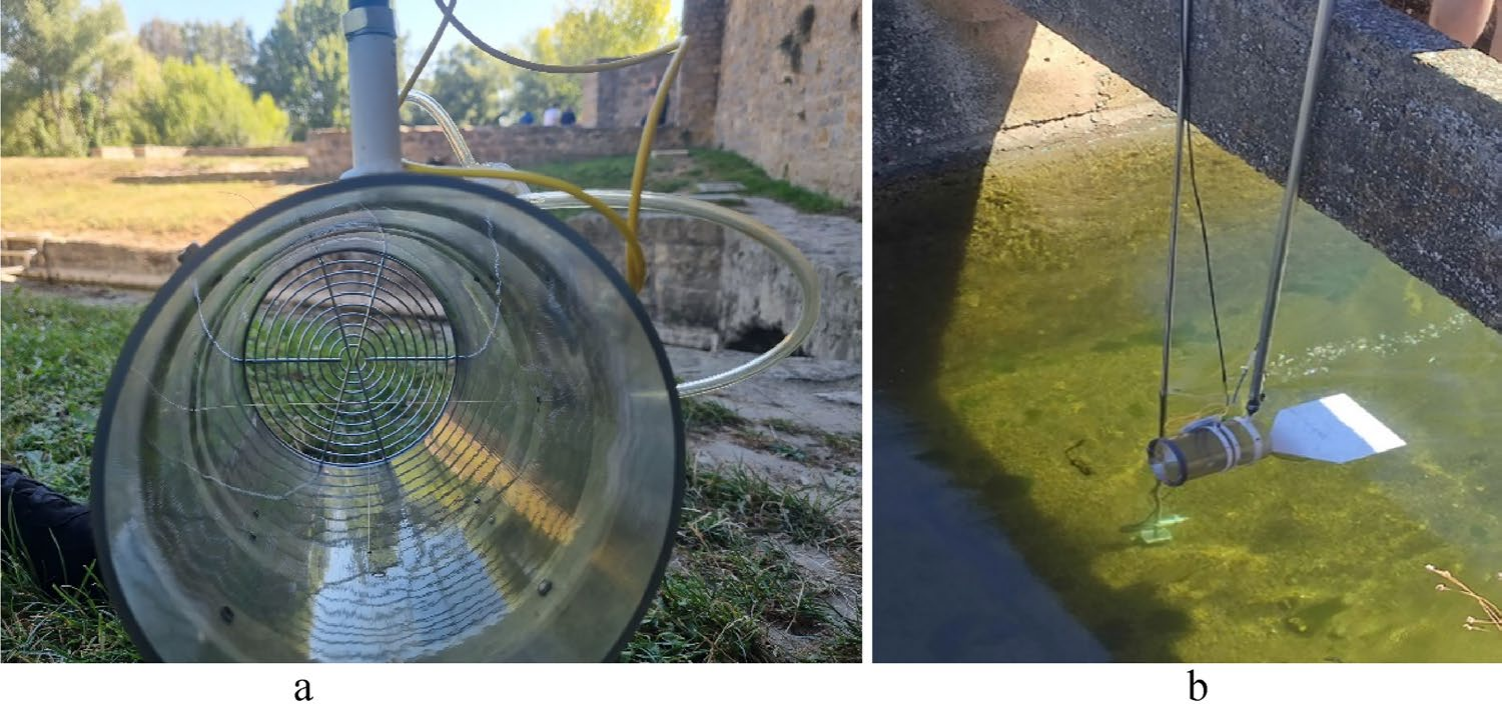
(**a**) Sensor holder for fiber probe, (**b**) Sensor setup placed in channel, next to the commercial flow meter magnetic probe.

Field testing was carried out with a hand-crafted probe for fiber optic sensor holding. This structure is intended as a proof of concept to demonstrate that it is possible to leave FBG sensors inside a channel or river for hours without deterioration. As shown in Fig. 8a), a circular pipe section acts as a guide for the water flow, causing the fiber optic wires to pass through it. For this purpose, two sensors with acrylate and aluminum coatings were placed in orthogonal positions.

An aluminum coating was selected to prevent small strands of dirt and floating objects from breaking the sensor. Mounting is performed by means of Teflon screws to avoid damage to the fiber and to ensure initial pretension during the entire measurement process. A metal grid at the front acts as a protection against residues present in the water, although small particles can stick themselves to the fibers, changing the surface geometry and, thus, the sensitivity. In this case, periodic maintenance helps to clean and recalibrate the sensors. A vane is placed at the back to achieve the orientation of the probe with the water stream.

The fiber optic sensor wires are also FBGs, with a diameter of 125 μm and a protective acrylate coating of 250 μm and another with a protective aluminum coating of 180 μm. This wire consists of the silica inner fiber (Young's modulus E = 70 GPa) and the polyimide coating (Young's modulus E = 2.76 GPa), whose coefficients have been determined by studying the elastic response of the fiber to various hanging masses and measuring the recovery capacity of the mechanical properties once they are removed^10^. The rigidity of acrylate is considered negligible due to its low Young's modulus; thus, *EA* = 860 N. The fiber ends are fastened at a distance of 0.16 m, and H = 0.08 m. The initial pretension is determined by a 20 g mass hanging from the end of the fiber and then secured using screws.

To compare and calibrate the sensors, an electronic water flow meter from OTT was used: OTT MF pro-Water Flow Meter, which shows an accuracy of ± 2% of the measured value, that is, ± 0.015 m s^−1^ from 0 to 3 m s^−1^. An electromagnetic water flow meter is a device that measures the flow velocity at a given point at a height above the base of the channel.

This device is based on Faraday’s law of electromagnetic induction ^17^. The water flow through the magnetic field generated by this equipment produces an electrical potential difference that is proportional to the velocity of the water. Once both sensors are placed, data registration is performed. The flow meter records the water velocity and depth, while for the OSI, the wavelength shifts from each sensor.

For the field test, four different scenarios were chosen: three open channels and a river. Figure 8b) displays the arrangement of both probes at the first visited site.

## Results and discussion

The flow rate via the experimental channel was manually increased to evaluate the sensor's performance. The canal had a 1% starting slope, and the height of the water level was also measured. Several iterations of this work were performed for statistical error analysis.

The average water velocity in Fig. 11 is calculated by applying the continuity Eq. ^18^

13 $$Q={V}_{m}\cdot A$$

where *Q* is the flow rate (m^3^ s^−1^); *V*_*m*_ is the average water velocity (m s^−1^); and *A* is the area of the section (m^2^).

**Figure 9:**
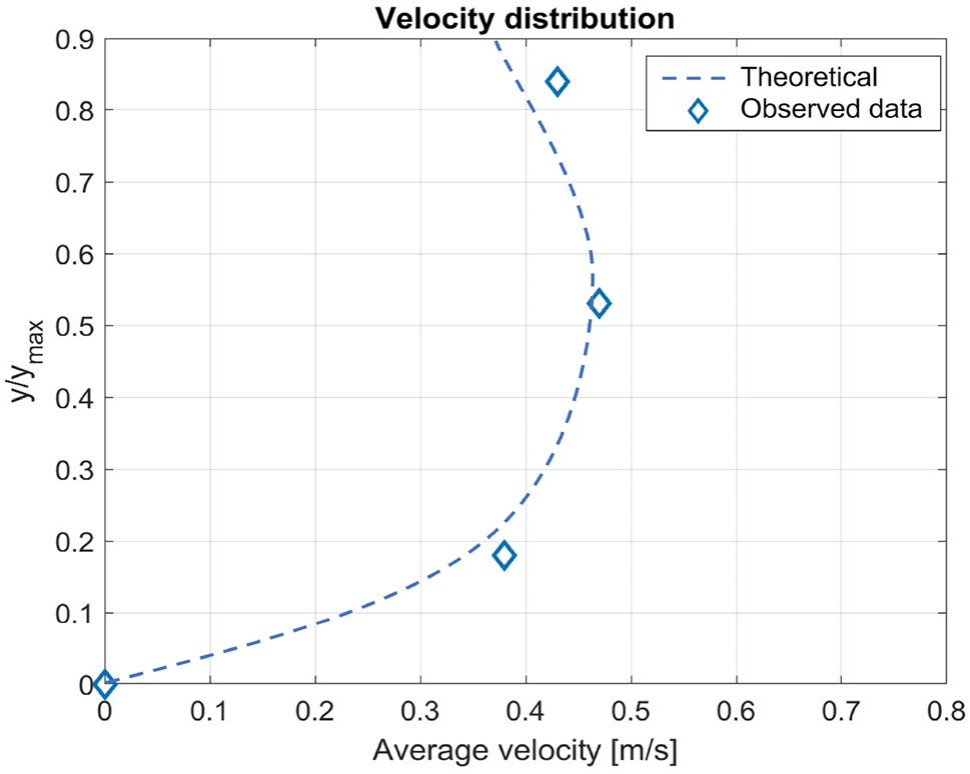
Velocity distribution at different heights obtained in the laboratory open channel.

Each grating is located in the middle of one of the three portions that make up the sensor head area. The average velocities calculated in each section contribute to the overall average velocity. After the analysis is complete, a velocity distribution curve is produced, as shown in Fig. 9. The trend highlights that the highest velocity values are attained in the core part of the channel and are closely related to the theoretical factors discussed previously.

To confirm the earlier findings and investigate how elevation affects water velocity, the procedure was repeated with a channel slope of 1.7%. Now that the upper sensor records the greatest value, it is clear that channel tilting causes an increase in surface currents.

**Figure 10:**
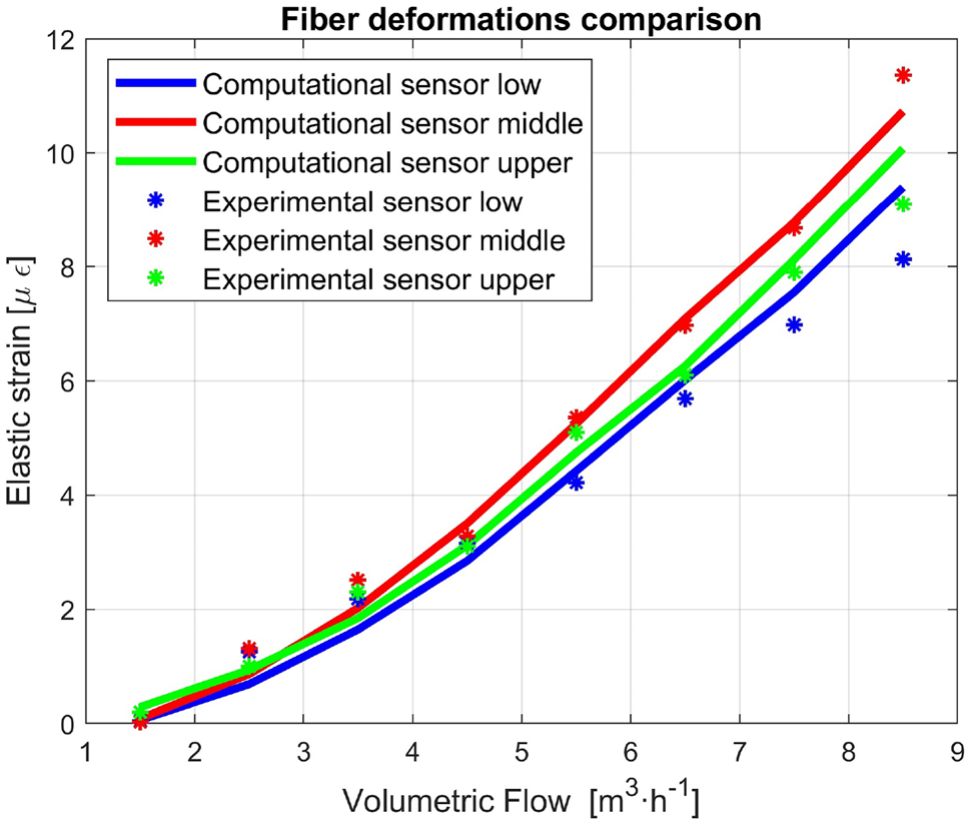
Comparative results of the measured strain vs simulations in this channel.

According to Fig. 10, the scenarios for the middle and lower sensors exhibit a linear trend that is consistent with the theoretical prediction for both the increase and fall of the flow rate. For this increased flow rate, an accurate approximation is observed.

14 $${V}_{m}=\frac{1}{n}{R}_{h}^{2/3}{S}^{1/2}$$

where *n*, Manning's uncertainty coefficient, characterizes the roughness of the channel.

*R*_*h*_: hydraulic radius, area in contact with the liquid.

*S*: canal slope.

Finally, the velocity distribution was also calculated by Manning’s formula as in Eq. (14). It is not feasible to use the continuity equation since the rate of change in the flow rate is the same as that in the previous test and the cross-sectional area is unchanged. Therefore, the slope causes the surface streams to be higher, and the wood block inside the channel stimulates the water to fall.

The simulations are in good agreement with the measured data.

As mentioned before, the measurements were also carried out in open channels and rivers by applying the initial pretension data calculated using Eq. (11). A total of twenty-six velocity values measured at different sites, with the corresponding optical fiber sensor strains, were measured in the tests.

The deformation measurements carried out for the first open channel are shown in Table 4. Row 1 is the water velocity (according to the electromagnetic flow meter), and rows 3 and 4 are the deformation measurements (elastic strain) for both coatings.

**Table 4:** Strain and water velocity recorded data.

	Measurements carried out in site 1				
V(m s^−1^)	0.87	0.96	1.08	0.77	0.91
ε (με)Acrylate	148	171	217	110	153
ε (με) Aluminum	102	123	158	79	112

**Figure 11:**
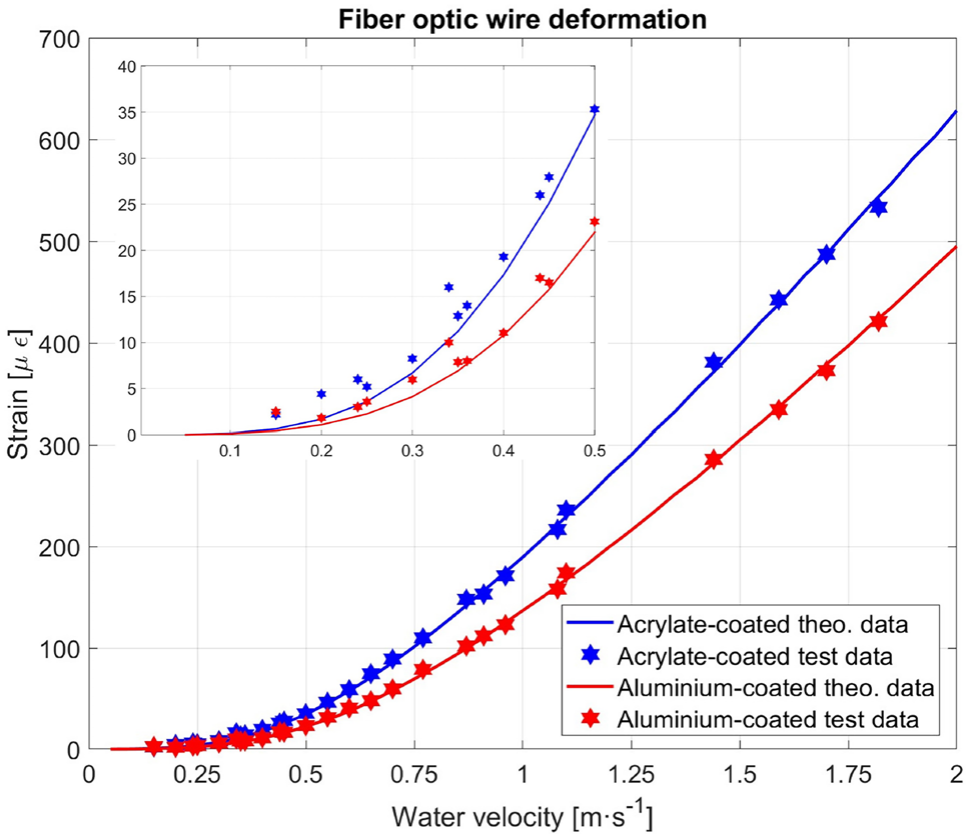
Theoretical strain versus measured strain (test) as a function of the water velocity and the coating material. The inset shows the low-velocities measurement zone.

To assess the repeatability of the experiment, four different places were visited, as mentioned before. Figure 11 shows the predicted strain included in the theoretical calculation (column 6 of Table 2) and the experimental measures of the strain (partially shown in rows 2 and 3 of Table 4) against the water velocity shown on the horizontal axis. In addition, the data recorded from the other places are included in that figure. An amplification of the low-speed zone allows us to emphasize the good approximation of the experimental procedure to the theoretical data.

Additionally, in this figure, the theoretical strain data calculated for both coatings are shown as solid lines. The markers indicate the recorded experimental data. The root mean square errors (RMSs) between the theoretically calculated data and experimental data are 2.827 µε for aluminum and 4.176 µε for acrylate.

**Figure 12:**
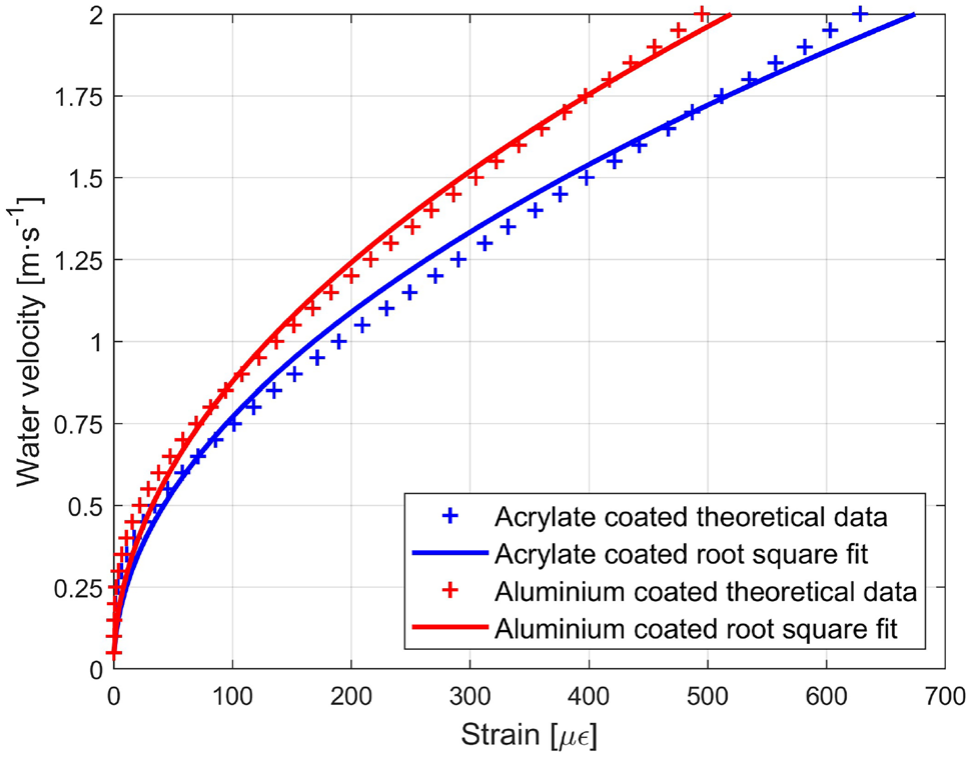
Theoretical strain versus root square fit of the strain.

An approximation for the theoretical values, which have shown excellent correlation with the experimental values, can be obtained by using a parabolic or square root fit. The parabolic fit $y=a{x}^{2}$ (where *y* is the strain and *x* is the water velocity) gives the following values for the $a$ coefficient: 129.91 for the aluminum coating and 168.60 for the acrylate coating. The obtained RMSs were 18.218 µε for the acrylate-coated fiber and 9.854 µε for the aluminum-coated fiber. The square root fit $y=b\sqrt{x}$ (*y* water velocity, *x* strain) gives a *b* coefficient of 0.0763 for acrylate and 0.0877 for the aluminum coating. Figure 12 shows the theoretical values and their respective square root fits $y=b\sqrt{x}$. This demonstrates the ease of calibration of the optical fiber sensors used.

The hand-crafted probe perfectly protected both sensors during the measurements. However, more secure new designs are being prepared for long-term measurements of rivers, preventing the effects of different detritus, water turbulence, and aquatic life. Additionally, tens of FBG-based sensors can be simultaneously interrogated with only an FBG interrogator (11). These sensors can be placed at different positions into the channel or river or at different heights at the same location. Currently, if few sensors are needed, modern integrated optics FBGs interrogators can offer a price that is one-third that of some electromagnetic flowmeters. Additionally, fiber optic cables that are perfectly protected for hostile environments (water, rodents) are commercially available and easily available.

## Conclusions

The FBG sensors have proven to be effective instruments for water management and distribution systems because they can offer precise measurements of water velocity and distribution in open channel environments. The FBG sensors used here are smaller in size than the other sensors currently in use, so they do not alter the flow of water even in narrow channels. Additionally, because optical fibers are dielectric, they are well suited for immersion in water.

The findings of this study demonstrate that FBG sensors have potential for real-time in-field measurements (open channels and rivers). It has demonstrated that both aluminum and acrylate coatings for FBGs can be used in real measurements and the ease of calibration of the optical fiber sensors used.

These results offer a novel method for precise, nonintrusive and effective monitoring of water velocity in real applications, which can be useful for scientists and engineers studying water management and distribution.

## Data Availability

The authors declare that the data supporting the findings of this study are available on request. Inquiries should be addressed to the corresponding author at armando.rodriguez@unavarra.es.
